# VirB10 vaccination for protection against *Anaplasma phagocytophilum*

**DOI:** 10.1186/s12866-018-1346-x

**Published:** 2018-12-18

**Authors:** Francy L. Crosby, Anna M. Lundgren, Carol Hoffman, David W. Pascual, Anthony F. Barbet

**Affiliations:** Department of Infectious Diseases and Immunology, College of Veterinary Medicine, University of Florida, 2015 SW 16th Avenue, Gainesville, FL 32608 USA

**Keywords:** *Anaplasma phagocytophilum*, Gram-negative bacterium, T4SS, VirB10 vaccination, DNA vaccine, Recombinant protein vaccine, Prime-boost immunization

## Abstract

**Background:**

Human granulocytic anaplasmosis (HGA) is a tick-borne disease caused by the etiologic agent *Anaplasma phagocytophilum*. HGA was designated a nationally notifiable disease in the United States in 1998. Currently there are no vaccines available against HGA. Conserved membrane proteins that are subdominant in *Anaplasma* species, such as VirB9 and VirB10, may represent better vaccine targets than the variable immunodominant surface proteins. VirB9 and VirB10 are constituents of the Type 4 secretion system (T4SS) that is conserved amongst many intracellular bacteria and performs essential functions for invasion and survival in host cells.

**Results:**

Immunogenicity and contribution to protection, provided after intramuscular vaccination of plasmid DNA encoding VirB9-1, VirB9-2, and VirB10 followed by inoculation of homologous recombinant proteins, in a prime-boost immunization strategy was evaluated in a murine model of HGA. Recombinant VirB9-1-, VirB9-2-, and VirB10-vaccinated mice developed antibody responses that specifically reacted with *A. phagocytophilum* organisms. However, only the mice vaccinated with VirB10 developed a significant increase in IFN-γ CD4^+^ T cells and partial protection against challenge with *A. phagocytophilum*.

**Conclusions:**

This work provides evidence that *A. phagocytophilum* T4SS VirB10 is partially protective in a murine model against infection in an IFN-γ-dependent fashion and suggests that this protein may be a potential vaccine candidate against this and possibly other pathogenic bacteria with a T4SS.

## Background

The order Rickettsiales, of which *A. phagocytophilum* is a member, includes numerous pathogens of humans and animals and agents requiring high containment because of the risk of spread. Many of these infections are difficult to diagnose because of non-specific disease symptoms. Few effective vaccines are available for this group of organisms, although strong immunity can develop in humans and animals that have been infected and recovered [1]. Vaccination has been problematic because of strain or antigenic variation of immunodominant outer membrane proteins. It has been proposed that better vaccine targets are subdominant, conserved proteins that have not been the primary targets of immune selection [2–5]. Components of the Type 4 Secretion System (T4SS) make good vaccine candidates due to their subdominant nature [6, 7]. The T4SS is a multiprotein complex that forms membrane channels required for the translocation of virulent substrates that enable colonization and survival of bacteria in the host cells. Although all Rickettsiales have a T4SS [8], immune responses against these components have been most extensively investigated in the bovine pathogen *Anaplasma marginale* [6, 9–15]. Cattle vaccinated with *A. marginale* outer membranes are protected against infection and respond strongly to membrane protein components. These responses are made against immunodominant, antigenically variable proteins as well as against VirB9 and VirB10 components of the T4SS [13]. Outer membrane protein vaccination of cattle induces strong IgG2 and CD4^+^ T cell proliferative and IFN-γ responses against VirB9 and VirB10 [6, 9]. Recently, a study tested the immunogenicity of recombinant *A. marginale* VirB9-1 and VirB9-2 in C57BL/6 J mice and indicated that these proteins also elicited strong humoral and T-cell mediated responses [16].

Sera from dogs experimentally infected with *Ehrlichia canis* reacted with recombinant VirB9, demonstrating that this protein is also immunogenic in organisms of this species [17]. There is evidence that in Rickettsiales VirB9 may be surface-exposed on outer membranes [18, 19]. A comparative genomics study of seven strains of *A. phagocytophilum* was recently done showing that VirB9 and VirB10 are conserved between strains, unlike other T4SS components such as VirB2 and VirB6. There are two VirB9 paralogs, VirB9-1 and VirB9-2, both notably having predicted signal peptides. Of all the potentially exposed components of the T4SS, VirB9 and VirB10 are the least diverse among strains [18]. Herein, we queried whether soluble recombinant protein analogs of VirB9 and VirB10 might confer protective immunity against *A. phagocytophilum* in a mouse model.

## Results

### Preparation of soluble recombinant VirB9-1, VirB9-2 and VirB10 proteins for immunization

**Fig. 1:**
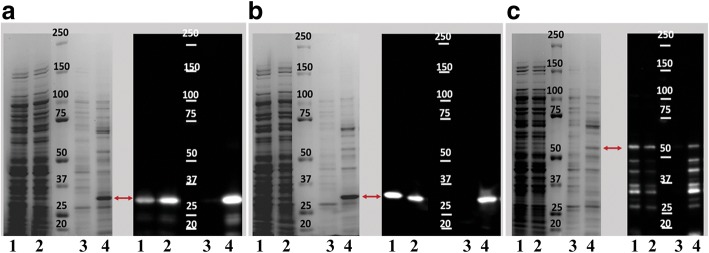
Analysis of the recombinant proteins VirB9-1, VirB9-2 and VirB10. SDS-PAGE and Western Blot analysis of recombinant VirB9-1 (**a**), VirB9-2 (**b**), and VirB10 (**c**). Lanes represent the different fractions analyzed during the purification procedure, 1 = Total crude protein, 2 = Filtered supernatant fraction obtained after high-speed centrifugation, 3 = Washed fraction, 4 = Eluted protein. Western Blot analysis was performed using the monoclonal anti-histidine tag antibody reacting with the recombinant protein (red arrows). Predicted molecular weights plus tags for VirB9-1 (33.5KDa), VirB9-2 (31.6KDa) and VirB10 (52KDa)

Recombinant VirB9-1, VirB9-2, and VirB10 were cloned and expressed as his-tag fusions in *E. coli.* We developed a method that resulted in the production of recombinant antigens that did not require purification from inclusion bodies or additional solubilization and refolding steps that possibly might affect the recovery of a functional protein. Soluble fractions of recombinant VirB9-1, VirB9-2, and VirB10 before and after nickel column purification were analyzed by Coomassie blue-stained SDS-PAGE gels, and the expected sizes plus his-tags were confirmed by Western blot (Fig. 1). These optimized culture conditions resulted in the production of adequate yields of soluble recombinant proteins suitable for the vaccination experiments. The recombinant proteins were of the sizes expected, with the exception of VirB10 where there were a series of fragments reactive with anti-his tag antibody up to and including the expected size of 52KDa. These may represent proteolytic degradation fragments of VirB10.

### *Anaplasma phagocytophilum* VirB9-1, VirB9-2, and VirB10 induce elevated antibody titers in mice

Five groups of mice were primed with the individual eukaryotic DNA expression plasmids encoding *A. phagocytophilum* VirB9-1, VirB9-2, or VirB10, their combination, or empty *pcDNA3.1* vector followed by boost with their respective recombinant proteins, or for the negative control, ovalbumin. First, serial dilutions of pooled sera from the three mice per group sacrificed before challenge were tested by IFA using *A. phagocytophilum*-infected HL-60 cells as a source of antigen. Red fluorescent inclusions corresponding to vacuoles filled with *A. phagocytophilum* organisms (morulae) were detected using sera from all VirB-vaccinated but not from control mice (data not shown).

**Fig. 2:**
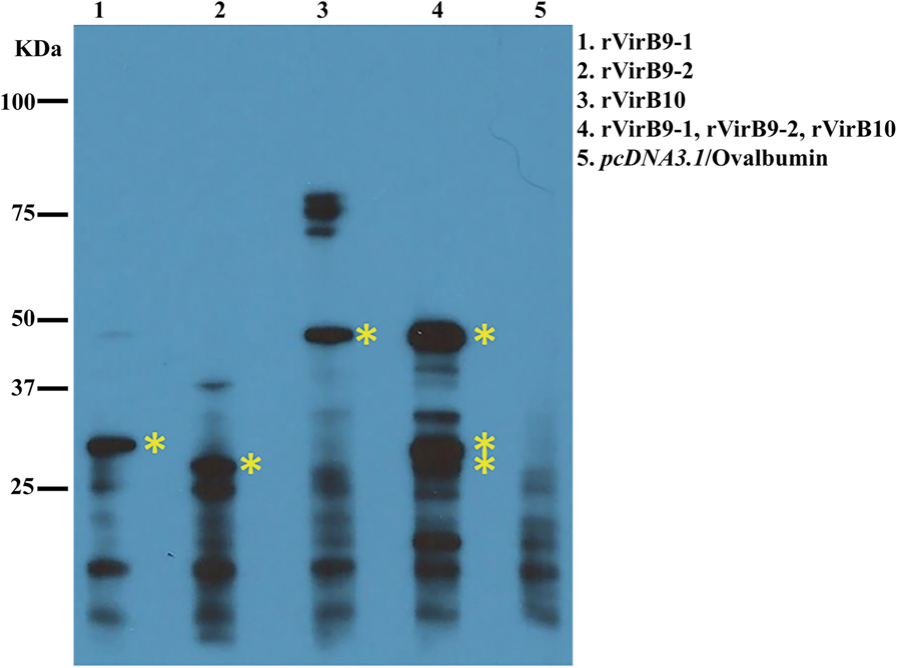
Sera from C3H/HeN immunized mice reacted against *A. phagocytophilum* VirB9-1, VirB9-2, and VirB10 proteins. Proteins from equal amounts (10^8^) of host cell-free *A. phagocytophilum* were separated by SDS-PAGE gel electrophoresis. Immunoblots of transferred proteins were reacted with pooled sera from the 3 immunized but not challenged mice (1:1000) and reactions were visualized by chemiluminescence. The sizes of protein standards are indicated on the left in kDa. Predicted molecular weights for *A. phagoytophilum* VirB9-1 (30.5KDa), VirB9-2 (28.6KDa), and VirB10 (49.2KDa). Bands of similar sizes were not visualized in sera from the *pcDNA3.1*/Ovalbumin control group

These sera were also assayed by Western blot of proteins from equal amounts of host cell-free *A. phagocytophilum* organisms (Fig. 2). Results show that sera from mice vaccinated with rVirB9-1, rVirB9-2, rVirB10, and (rVirB9-1-rVirB9-2-rVirB10) mix reacted with proteins of molecular sizes that corresponded to *A. phagocytophilum* VirB9-1, VirB9-2, and VirB10. In contrast, (*pcDNA3.1*/Ovalbumin)-vaccinated mice did not develop antibodies against the same size proteins.

**Fig. 3:**
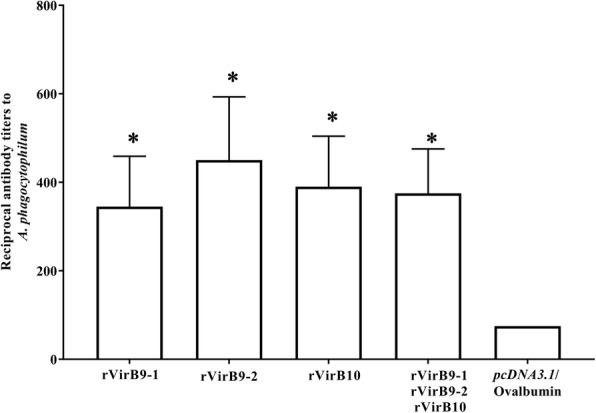
Anti-*A. phagocytophilum* antibody titers. Sera from all 10 immunized and control mice in each group were collected before challenge at two weeks after the last immunization. Endpoint titers were evaluated by ELISA. ^*^, *P* < 0.05

We used ELISA to quantify *A. phagocytophilum* VirB9-1, VirB9-2 and VirB10-specific antibodies in sera from all 10 vaccinated mice in each group before challenge. As antigen, we used host cell-free *A. phagocytophilum* organisms (10^8^) applied to each well of ELISA plates. Vaccination with rVirB9-1, rVirB9-2, rVirB-10 and (rVirB9-1-rVirB9-2-rVirB10) mix induced the production of significantly higher antibody titers against *A. phagocytophilum* organisms relative to (*pcDNA3.1*/Ovalbumin)-vaccinated mice (Fig. 3). The endpoint mean titers based on this assay were (Mean ± SEM) 345 ± 114.1, 450 ± 143.1, 390 ± 114.4 and 375 ± 100.6 in rVirB9-1, rVirB9-2, rVirB10 and (rVirB9-1-rVirB9-2-rVirB10)-vaccinated mice respectively.

### VirB10 confers partial protection against *A. phagocytophilum* challenge

**Fig. 4:**
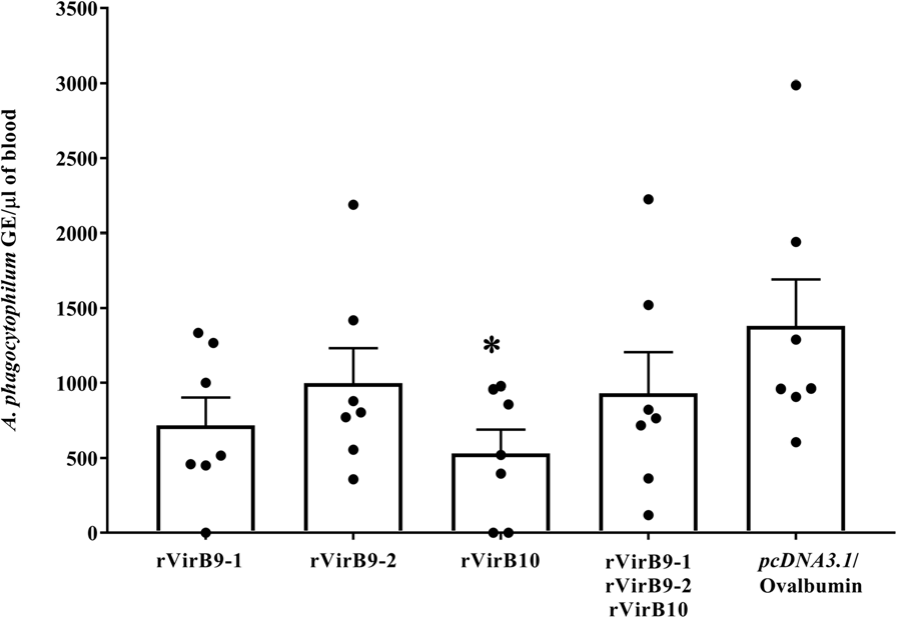
Immunization with rVirB10 confers partial protection against *A. phagocytophilum* challenge. *A. phagocytophilum* GE means at the peak of infection (day 8 post-challenge). Each symbol represents the value for an individual mouse. ^*^, *P* < 0.05

To evaluate protective efficacy of rVirB9-1, rVirB9-2, rVirB10 and the mixture of the three subunits against *A. phagocytophilum* challenge, we infected rVirB-vaccinated and irrelevant antigen-vaccinated mice (7/group) with one dose of isolated organisms from 5.63 × 10^5^ HL-60 infected cells (90% of the cells contained morulae). Bacteria loads in blood following challenge were quantitated by real time qPCR assay targeting the single copy gene *msp5* to determine the number of *A. phagocytophilum* genome equivalents (GE). This assay detected maximum bacterial loads in all groups at day 8 post infection. However, only in the group of mice vaccinated with rVirB10, bacteria replicated to significantly lower levels (Mean ± SEM peak bacterial load of 530 ± 159.9, *P* = 0.032) than in the (*pcDNA3.1*/Ovalbumin)-vaccinated mice (Mean ± SEM peak bacterial load of 1380 ± 311.6) (Fig. 4). Moreover, two mice from the rVirB10 group cleared the infection, as we did not detect *A. phagocytophilum* at any time post-challenge. Although the bacterial loads in the groups of mice vaccinated with rVirB9-1, rVirB9-2 and the (rVirB9-1-rVirB9-2-rVirB10) mix were lower than the control group, this reduction was not significant.

To assess the correlation between anti-*A. phagocytophilum* VirB9-1, VirB9-2 and VirB10 antibody titers produced before challenge and protection against *A. phagocytophilum*, the ELISA endpoint titers were plotted as a function of the bacteria loads. Pearson’s correlation tests for all 7 vaccinated and challenged mice in each group showed no correlation between reduced bacterial loads and antibody titers.

### VirB10 induces CD4^+^ Th1 immune responses in vaccinated mice

**Fig. 5:**
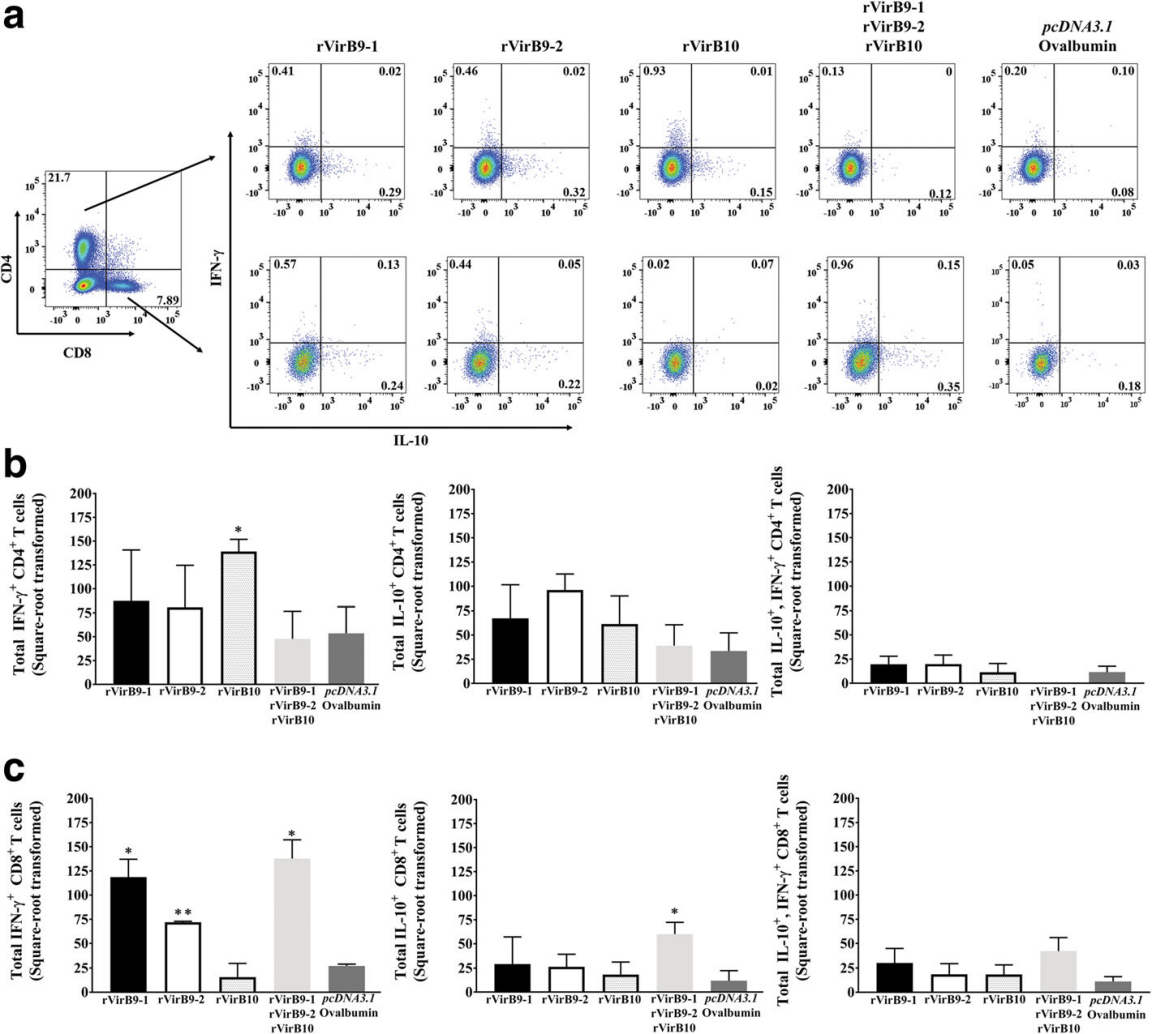
CD4^+^ and CD8^+^ T cell responses from rVirB-vaccinated mice before *A. phagocytophilum* challenge. Spleen lymphocytes from rVirB and control (*pcDNA3.1*/Ovalbumin)-vaccinated mice were evaluated for intracellular IFN-γ^+^ and IL-10^+^ production by flow cytometry. **a** representative plots, gated for CD4^+^ and CD8^+^ T cells, depicting IFN-γ^+^, IL-10^+^, and double-positive (IL-10^+^-IFN-γ^+^) T cells. **b** and (**c**) bar graphs displaying total IFN-γ^+^, IL-10^+^, and double-positive (IL-10, IFN-γ^+^)CD4^+^ and CD8^+^ T cells. ^*^, *P* = 0.01 to 0.05; ^**^, *P* = 0.001 to 0.01

To evaluate T cell responses to total *A. phagocytophilum* proteins, as source of VirB9-1, VirB9-2 and VirB10, splenic lymphocytes from the different vaccination groups were analyzed by flow cytometry before and after challenge. Before challenge, the rVirB10-vaccinated mice were the only group that stimulated antigen-specific CD4^+^ T cell responses that were characterized by 2.6-fold higher numbers of IFN-γ^+^ CD4^+^ T cells than the irrelevant antigen (*pcDNA3.1*/Ovalbumin)-vaccinated mice (Fig. 5a, b). No significant differences in the numbers of IL-10^+^ and double-positive (IL-10^+^-IFN-γ^+^) CD4^+^ T cells in any of the rVirB-immunized mice compared to the (*pcDNA3.1*/Ovalbumin)-vaccinated mice were observed. In contrast, rVirB9-1, rVirB9-2 and (rVirB9-1-rVirB9-2-rVirB10)-vaccinated mice, but not those vaccinated with rVirB10, stimulated CD8^+^ T cells. This was indicated by 4.36, 2.7 and 5.09-fold increase in the numbers of IFN-γ^+^ CD8^+^ T cells in mice vaccinated with rVirB9-1, rVirB9-2, and the (rVirB9-1-rVirB9-2-rVirB10) mix, respectively, when compared to (*pcDNA3.1*/Ovalbumin)-vaccinated mice (Fig. 5c). Likewise, mice vaccinated with the (rVirB9-1-rVirB9-2-rVirB10) mix had 5.0-fold higher numbers of IL-10^+^ CD8^+^ T cells relative to (*pcDNA3.1*/Ovalbumin)-vaccinated mice (Fig. 5c). So, vaccination with rVirB10 induced IFN-γ^+^ CD4^+^ T cells while vaccination with rVirB9-1, rVirB9-2 and these two in combination with rVirB10 induced IFN-γ^+^ or IL-10^+^ CD8^+^ T cells.

**Fig. 6:**
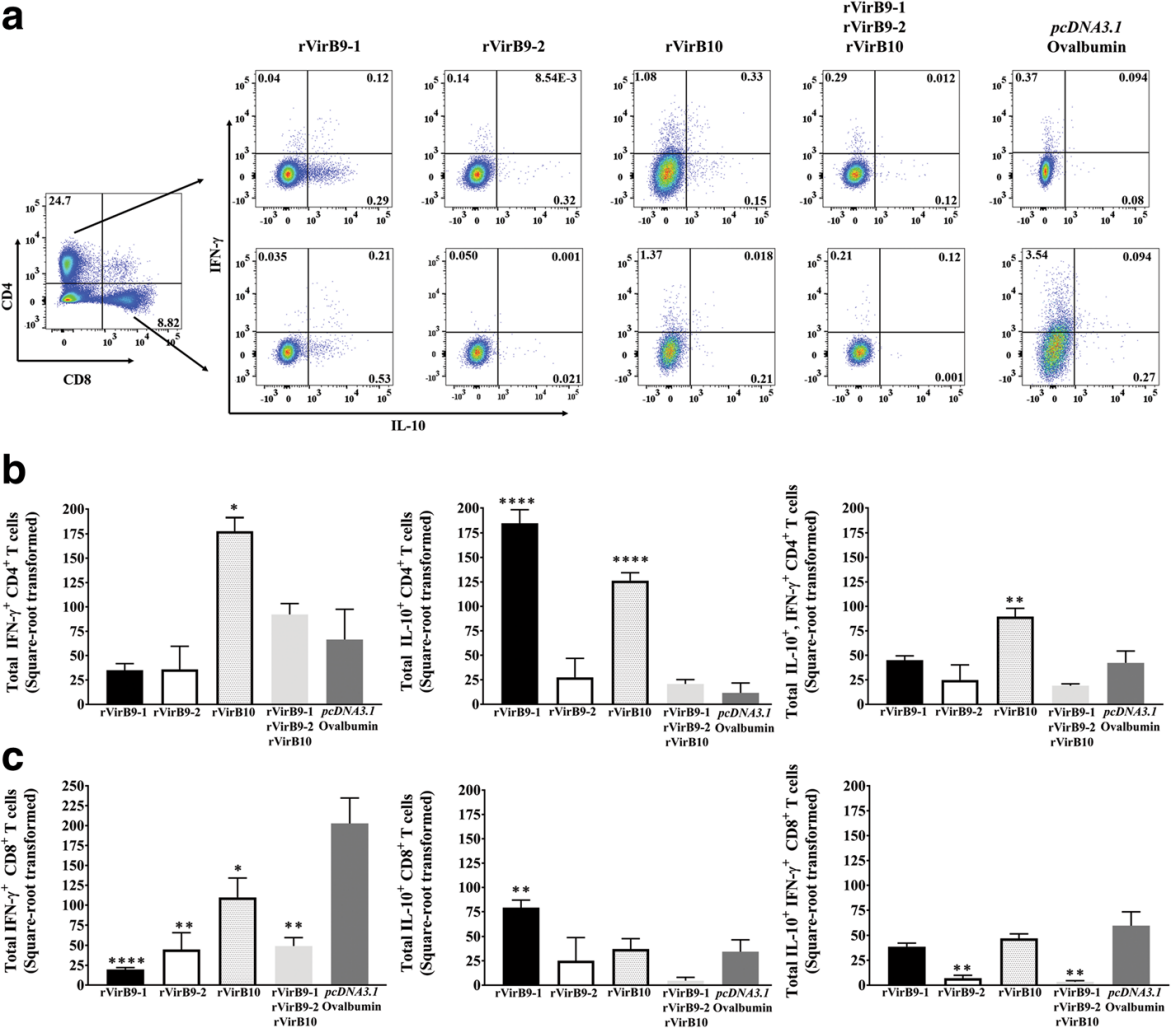
CD4^+^ and CD8^+^ T cell responses from rVirB-vaccinated mice after *A. phagocytophilum challenge*. Spleen cells from rVirB and control (*pcDNA3.1*/Ovalbumin)-vaccinated mice were evaluated for intracellular IFN-γ^+^ and IL-10^+^ production by flow cytometry. **a** representative plots, gated for CD4^+^ and CD8^+^ T cells, depicting IFN-γ^+^, IL-10^+^, and double-positive (IL-10^+^-IFN-γ^+^) T cells. **b** and (**c**) bar graphs displaying total IFN-γ^+^, IL-10^+^, and double-positive (IL-10^+^, IFN-γ^+^) CD4^+^ and CD8^+^ T cells. ^*^, *P* = 0.01 to 0.05; ^**^, *P* = 0.001 to 0.01; ^****^, *P* < 0.0001

After *A. phagocytophilum* challenge, distinct antigen-specific T cell responses were observed mainly in rVirB9-1 and rVirB10-vaccinated mice. Again, mice vaccinated with rVirB10 displayed better CD4^+^ T cell responses to total bacterial antigens, not only by maintaining elevated numbers (2.7-fold) of IFN-γ^+^ CD4^+^ T cells, but also with a 10.9 and 2.1-fold expansion in the numbers of IL-10^+^ and double-positive (IL-10^+^-IFN-γ^+^) CD4^+^ T cells, respectively, when compared to (*pcDNA3.1*/Ovalbumin)-vaccinated mice (Fig. 6a, b). Contrary to that observed before challenge, mice vaccinated with rVirB9-1 also stimulated CD4^+^ T cells as evidenced by 15.6-fold greater numbers of IL-10^+^ CD4^+^ T cells relative to the (*pcDNA3.1*/Ovalbumin)-vaccinated mice (Fig. 6b). Interestingly, the IL-10^+^ CD4 T cell numbers from rVirB9-1-vaccinated mice exceeded IFN-γ^+^ CD4 T cell numbers from the same group by 5.26–fold (*P* < 0.0001). However, IFN-γ^+^ CD4^+^ T cell numbers from rVirB10-vaccinated mice were 1.4-fold (*P* < 0.01) above the same group’s IL-10^+^ CD4^+^ T cell numbers.

Conversely, the numbers of antigen-specific IFN-γ CD8^+^ T cells were significantly reduced by 10.3, 4.5, 1.8 and a 4.8-fold in rVirB9-1, rVirB9-2, rVirB10 and (rVirB9-1-rVirB9-2-rVirB10)-vaccinated mice, respectively, compared to the (*pcDNA3.1*/Ovalbumin)-vaccinated mice (Fig. 6c). However, only rVirB9-1 vaccination induced significant numbers of IL-10 CD8^+^ T cells, with 2.3-fold higher numbers than in (*pcDNA3.1*/Ovalbumin)-vaccinated mice (Fig. 6c).

These results indicate that after *A. phagocytophilum* challenge rVirB10 continued to specifically stimulate CD4^+^ T cells through a better response characterized by elevated numbers of IFN-γ^+^, IL-10^+^ and double-positive (IFN-γ^+^-IL-10^+^) CD4^+^ T cells. Yet, rVirB9-1 induced a prominent IL-10 immune response in both CD4^+^ and CD8^+^ T cells, while the IFN-γ^+^ CD8^+^ T cell responses induced by rVirB9-1 and rVirB9-2 before challenge were abrogated.

## Discussion

There are several *A. phagocytophilum* strains with unique adaptations to different host species, pathogenicity, clinical presentation, and immunological responses [20, 21]. Despite their diversity, a common element in all *A. phagocytophilum* strains sequenced to date is the presence of genes encoding components of the T4SS prototypical to the VirB/VirD system of *Agrobacterium tumefaciens*. Comparative genomics of several *A. phagocytophilum* strains to define the structure of the T4SS identified that VirB9 and VirB10 are the most conserved [18]. VirB9 and VirB10 are also conserved among other species of the order Rickettsiales and although subdominant in natural infections, these proteins induce responses when outer membranes are used to immunize animals [6, 9–11, 13, 17]. Hence, their conserved nature and possible role in pathogenesis make them ideal candidates for vaccine development.

In this work, we tested the ability of recombinant VirB9-1, VirB9-2, and VirB10 (alone or combined) to stimulate protective immunity against *A. phagocytophilum* in C3H/HeN mice. We prepared DNA vaccine constructs encoding VirB9-1, VirB9-2, and VirB10 as well as their respective recombinant proteins, and used them in a prime-boost immunization regimen. This vaccination approach has proven to improve and broaden protective immune responses against a wide range of pathogens [22–28] including bacterial species of the order Rickettsiales, such as *Rickettsia conorii*, *Rickettsia rickettsii* [29]. *Ehrlichia ruminantium* [30], *Ehrlichia muris* [31], and *Anaplasma marginale* [32].

The antigenic structure of a recombinant protein does not always mimic that of the homologous protein in its native environment, especially of membrane proteins produced as insoluble inclusion bodies that require refolding and solubilization [33, 34]. We, therefore, prioritized maintaining solubility of the recombinant VirB’s during expression and purification over their yield. Considering the importance of assessing if a recombinant antigen retains epitopes similar to the native protein in the infective form of the pathogen [35–37], we aimed to evaluate humoral and cellular responses from recombinant VirB9-1, 9-2 and VirB10 vaccinated mice wherever possible directly against *A. phagocytophilum* organisms. This was done by using purified organisms from cell cultures as antigen on ELISA plates in antibody testing and as stimulating antigen in cellular immune response assays. This approach should limit the potential detection of responses against *E. coli* contaminants present in the immunizing recombinant antigens. However, we recognize the potential significance of *E. coli* contaminants and of VirB10 fragments (such as those seen in Fig. 1c) possibly contributing to immune stimulation. This requires further investigation and replication, particularly in outbred animal populations with more heterogeneous responses.

We evaluated antibody responses to rViB9-1, rVirB9-2, and rVirB10 and established that vaccinated mice developed elevated levels of antibodies that specifically reacted with native VirB9-1, VirB9-2, and VirB10 proteins. Yet, there was a lack of correlation between antibody titers against native VirB proteins from *A. phagocytophilum* and protection against infection. There is evidence that in Rickettsiales, antibodies bind to surface exposed antigens required for initial stages of infection (attachment to the host cell), or during intercellular spreading, to block infection [4, 38–40]. Also, BlastP searches showed significant homology between VirB10 of *A. phagocytophilum* and *E. coli* (E value of 3e-18, 32% identity). In *E. coli*, two α-helices in the C-terminal domain of VirB10 (the “antennae”) are exposed to the extracellular environment at the top of the T4SS pore [41]. Therefore, it is possible that vaccination elicited weak or very low levels of antibodies that bind to exposed or neutralization-sensitive epitopes to prevent infection of susceptible cells. Such anti-VirB10 antibodies might not be apparent in the ELISA used in Fig. 3 which assayed for total organism-binding antibodies. Possibly, predicted extracellular domains of *A. phagocytophilum* VirB10, could be included in the design of epitope-based vaccines also targeting other surface exposed molecules such as outer membrane protein A, Asp14 and AipA [39, 40, 42, 43].

Examination of the induced T cell responses to these different VirB proteins revealed a dichotomy in their ability to elicit IFN-γ responses. IFN-γ is a Th1 cell-promoting factor important for immune protection against intracellular pathogens including *A. phagocytophilum* [44–46]. Prior to challenge, mice vaccinated solely with VirB10 induced an elevated IFN-γ^+^ CD4^+^ T cell response following restimulation with whole *A. phagocytophilum* as a source of VirB10. In contrast, the same group of mice showed a weak IFN-γ^+^ CD8^+^ T cell response suggesting that the dominant immunogenic peptides for VirB10 are recognized by CD4^+^ T cells. Mice vaccinated with rVirB9-1, rVirB9-2, or both proteins combined with rVirB10 showed no significant differences in their IFN-γ^+^ CD4^+^ T cells from vector control mice prior to challenge. However, the same was not true for their respective CD8^+^ T cell responses since both rVirB9-1- and rVirB9-2-immunized groups showed significantly greater IFN-γ^+^ CD8^+^ T cells than vector control or rVirB10-immunized mice. In fact, the mice immunized with all 3 proteins also showed an elevated IFN-γ^+^ CD8^+^ T cell response implicating the contributions by rVirB9-1 and rVirB9-2 immunogens.

In mice infected with *A. phagocytophilum*, CD4^+^ T cells are critical for the clearance of bacteria [44, 47]. Several studies have shown that, during the initial stages of *A. phagocytophilum* infection, IFN-γ-deficient mice or IFN-γ receptor-deficient mice developed increased bacterial loads in blood and tissues compared to immunocompetent mice, indicating the importance of this cytokine in the control of bacteremia [44, 47, 48]. Our results show that following *A. phagocytophilum* challenge, only mice vaccinated with recombinant VirB10 were protected, as evidenced by the significant reduction in the *A. phagocytophilum* burden. Such protection was associated with increased levels of IFN-γ^+^ CD4^+^ T cells induced by VirB10 since mice immunized with rVirB9-1, rVirB9-2, or all three proteins did not have significant reductions in their *A. phagocytophilum* burdens nor showed elevations in their IFN-γ^+^ CD4^+^ T cells. In contrast, the CD8^+^ T cell responses elicited by rVirB9-1, rVirB9-2, or all three proteins resulted in reduced IFN-γ^+^ cells. Why rVirB9-1 and rVirB9-2 were less effective immunogens may be tied to their ability to stimulate IL-10 either in preference to IFN-γ or to regulate IFN-γ responses. IL-10 is associated with immunoregulation of the host response dampening inflammatory responses including IFN-γ [49], and there is evidence that during HGA IL-10 has an anti-inflammatory role [21, 48, 50].

The present work demonstrates stimulation of different host responses, either CD4^+^ or CD8^+^ T cell-dependent and their uniquely associated cytokines. In this regard, while none of the groups showed a predilection for IL-10 production by the induced CD4^+^ or CD8^+^ T cells prior to challenge, the rVirB9-1-vaccinated mice showed the greatest IL-10^+^ CD4^+^ and CD8^+^ T cell responses subsequent to challenge. This IL-10^+^ CD4^+^ T cell response was greater than the one induced in rVirB10-vaccinated mice. Therefore, IL-10 seems to dampen the induced, elevated IFN-γ^+^ CD4^+^ T cell response, which is induced by vaccination with VirB10 alone. This is further suggested by the increased presence of double-positive (IFN-γ^+^ IL-10^+^) CD4^+^ T cells. Double-positive (IL-10^+^-IFN-γ^+^) CD4^+^ T cells have been found during chronic infections caused by other intracellular pathogens such as *Leishmania* spp., *Trypanosoma* spp., *Mycobacterium tuberculosis*, and *Borrelia burgdorferi* and although their presence might contribute to persistence, it could also prevent collateral immune damage [51].

Studies of *A. phagocytophilum* infections in humans, mice and horses have shown that IFN-γ is also implicated in the development of tissue lesions due to overactive inflammatory responses, showing a dual role for this cytokine [44, 48, 52–54]. However, it is also known that the anti-inflammatory effect of IL-10 helps to decrease pathology during HGA [21, 48, 53]. For example, IFN-γ-deficient mice infected with *A. phagocytophilum* had higher bacterial burdens in blood and tissues than IL-10-deficient and parental wild-type mice but minimal histopathological lesions, while *A. phagocytophilum* infected IL-10-deficient mice developed severe histopathological lesions [48]. In humans with HGA, mild clinical manifestations and recovery from infection is temporally associated with a dominant IFN-γ immune response and moderate levels of IL-10 [48, 55]. Lastly, treatment of horses infected with *A.phagocytophilum* with the anti-inflammatory glucocorticoid dexamethasone resulted in an increased IL-10:IFN-γ ratio and higher bacterial loads than untreated animals, but reduced disease severity [53].

An adequate balance between pro- and anti-inflammatory responses may well result in an effective control against infection while restricting tissue damage [56]. Our results show that VirB10-induced production of IFN-γ and IL-10 might modulate the balance between immunopathological responses and control of infection. Collectively, these data show the immunogenicity of VirB10 favors protection while immunity to VirB9-1 and VirB9-2 failed to stimulate significant IFN-γ responses and may not be important for protection.

Intriguingly, only VirB9-1, VirB9-2, and in combination with VirB10 induced increased IFN-γ-producing CD8^+^ T cells in immunized mice before challenge. However, after challenge, these levels significantly dropped relative to the control suggesting that these may be inhibited during the challenge. The importance of CD8^+^ T cells for protection against *A. phagocytophilum* may be less. It has been previously shown that CD8^+^ T cells were not essential for controlling *A. phagocytophilum* infection because MHC class I-deficient mice were as competent in eliminating the pathogen as wild-type mice [47, 54, 57]. Rather, CD4^+^ T cells are necessary in mice for effective control of *A. phagocytophilum* infection because in the absence of MHC class II-restricted CD4^+^ T cells they failed to clear *A. phagocytophilum* [47, 54, 57].

## Conclusions

In conclusion, we show that prime-boost vaccination with VirB10, a subdominant conserved antigen that is a component of the T4SS, not only induced antigen-specific humoral and T cell mediated responses, but also elicited partial protection against *A. phagocytophilum* challenge.

## Methods

### Preparation of DNA vaccine constructs

The open reading frames of *A. phagocytophilum virB9-1*, *virB9-2* (devoid of their signal peptides), and *virB10* genes were amplified by PCR using the Taq DNA polymerase (ThermoFisher Scientific) and cloned into *pcDNA3.1*/CT-GFP-TOPO vector for high-level expression in mammalian hosts (ThermoFisher Scientific). The primers were designed to allow in frame translational fusions of these genes with the green fluorescent protein (GFP) at the C-terminal end under the regulation of the cytomegalovirus (CMV) promoter (Table 1).

**Table 1:** Oligonucleotides used in this study

		Target
DNA vaccines		
AB1656	GCCACCATGAGCACAAATATTGGCGTACC	*virB9-1*
AB1657	GACTAAGAGCCTGATTCACAACTTCTAC	
AB1658	GCCACCATGGCTGATGATCACCATTAAGACC	*virB9-2*
AB1659	GTTTCCGGCGTCTTTCAGCACCCTTC	
AB1660	GCCACCATGGCTGACGAAATAAGGGG	*virB10*
AB1661	GCCTCACCGCATCACGAGGAAATA	
Recombinant proteins		
AB1703	CACCATGAGCACAAATATTGGCGTACCAG	*virB9-1*
AB1704	ACTAAGAGCCTGATTCACAACTTCTACACTCCTGC	
AB1705	CACCATGGCTGATGATCACATTAAGACCTTGAAC	*virB9-2*
AB1706	TTTCCGGCGTCTTTCAGCACCCTTC	
AB1707	CACCATGGCTGACGAAATAAGGGGTTCTAG	*virB10*
AB1708	CCTCACCGCATCACGAGGAAATACTACG	
qPCR		
AB1334	AGATGCTGACTGGGGATGAG	*msp5*
AB1335	TCGGCATCAACCAAGTACAA	
^a^AB1336	CGTAGGTGAGTCTGATAGTGAAGG	

Underlined letters correspond to vector nucleotides added to enable directional cloning

^*a*^ *Probe labeled with Hexachloro-fluorescein (HEX) at the 5′ end*

Purified PCR products were ligated into *pcDNA3.1*/CT-GFP-TOPO vector and transformed into TOP10 *Escherichia coli* cells (ThermoFisher Scientific). Individual recombinant plasmids were analyzed by sequencing to determine the presence and correct orientation of inserts. Desired clones were transfected into RF/6A endothelial cells using Lipofectamine 2000 transfection reagent (ThermoFisher Scientific), according to the manufacturer’s instructions. Briefly, 0.5 μg of recombinant plasmid DNA in Opti-MEM reduced serum medium (ThermoFisher Scientific) was mixed with Lipofectamine 2000 and incubated for 5 min before adding to each well of a twenty four well plate containing nearly confluent cell monolayers. To evaluate transfection efficiency and GFP expression, *pcDNA3.1*/CT-GFP and mock DNA-lipofectamine mixture were used as positive and negative controls, respectively. At 48 h post-transfection, green fluorescent endothelial cells were visualized (data not shown) using a Leica DMI 3000B inverted microscope equipped with a GFP filter specific for excitation and emission wavelengths of 470/40 nm and 525/50 respectively.

### Preparation of recombinant VirB9-1, VirB9-2 and VirB10 protein vaccines

The open reading frames of *A. phagocytophilum virB9-1*, *virB9-2* (devoid of their signal peptide) and *virB10* genes were amplified by PCR using the iProof ™ High-Fidelity DNA polymerase (Bio-Rad). PCR primers were designed to incorporate specific vector sequences at the 5’end of the genes to enable directional cloning into the pET101/D-TOPO directional expression system (ThermoFisher Scientific) (Table 1). Purified PCR products were ligated into the pET101/D-TOPO vector and transformed into one shot TOP10 *E. coli* cells. Recombinant plasmids were recovered from individual colonies and *virB9-1*, *virB9-2,* and *virB10* in frame sequences were confirmed by PCR, restriction enzyme and sequencing analysis. Recombinant clones contained the genes of interest followed by V5 epitope and 6X His tags. These constructs were retransformed into *E. coli* BL21 Star (DE3) cells (ThermoFisher Scientific), and grown overnight at 37 °C in Luria-Bertani (LB) medium containing 1% glucose and 50 μg/mL carbenicillin until a cell density between OD_600_ of 3 to 5 was reached. Then, these cultures were switched to M9 minimal medium supplemented with glucose and carbenicillin at final concentrations of 1% and 50 μg/mL, respectively, and induced with 0.5 mM isopropyl β-D-thiogalactoside (IPTG) (Sigma-Aldrich) at 4 °C for an additional 28 h. We switched to M9 minimal medium in order to limit bacterial growth and the depletion of substrates and cofactors required for protein synthesis [58]. Expression was induced at 4 °C to minimize protein aggregation and to reduce heat shock protease activity that could result in the formation of inclusion bodies.

Bacterial cells were harvested by centrifugation, and pellets were disrupted by sonication in sodium phosphate buffer pH 8.0 (150 mM NaCl, 23.6 mM Na_2_HPO_4_, 1.2 M NaH_2_PO_4_) followed by centrifugation to fractionate the soluble and insoluble material. Soluble fractions containing the recombinant 6X His-tagged fusion proteins were purified using low density Nickel agarose bead columns (Gold Biotechnology). Eluted protein was concentrated using Centricon Plus-70 centrifugal filter units (EMD Millipore), and concentration determined using a Qubit protein assay kit (ThermoFisher Scientific). Verification of expression of soluble recombinant VirB9-1, Vir9-2, and VirB10 was performed by analysis of proteins on sodium dodecyl sulfate-polyacrylamide gel electrophoresis (SDS-PAGE) stained with GelCode Blue (ThermoFisher Scientific) and by Western blot analysis using a monoclonal mouse anti-His antibody (SignalChem).

### *Anaplasma phagocytophilum* cultivation

Two cell lines were used for this work: the HL-60 human promyelocytic cells and the RF/6A cells derived from the retina choroid endothelium of a rhesus monkey (*Macaca mulatta*). HL-60 cells were used to propagate the human isolate *Anaplasma phagocytophilum* strain HZ [59], and the RF/6A endothelial cells were used for transfection experiments as described above. Uninfected- and *A. phagocytophilum*-infected cultures were maintained in RPMI 1640 medium (Hyclone) supplemented with 10% heat inactivated fetal bovine serum (FBS) (HyCLone), 2 mM L-glutamine (ThermoFisher Scientific), 0.25% NaHCO_3_ (Sigma-Aldrich) and 25 mM HEPES (Sigma-Aldrich) and kept at 37 °C in 5% carbon dioxide (CO_2_) atmosphere.

### Preparation of host cell-free *A. phagocytophilum*

For analysis of immunoblots, ELISA, and T cell responses, *A. phagocytophilum* organisms purified from infected HL-60 cells were used. For this, heavily infected cultures were transferred into sterile 2.0 ml bead beater tubes (BioSpec Technologies) containing 1 mm diameter glass beads (BioSpec Technologies). Disruption of infected cells was performed in a Mini-Beadbeater (BioSpec Technologies) at 4800 rpm for 10s. Cell lysates were transferred to 1.5 ml tubes and centrifuged at 100 x g for 5 min at 4 °C to pellet cell debris. The supernatant was then carefully removed and filtered by passing it through a 2.0 μm glass fiber syringe filter (Whatman). Bacteria were then pelleted by centrifugation at 11,000 x g for 10 min at 4 °C and stored at − 20 °C for further protein analysis work, DNA extraction and quantitative real-time PCR (qPCR) for quantitation of *A. phagocytophilum* GE.

### Experimental animals, immunization, and challenge

Six-week-old female C3H/HeN mice were purchased from Charles River Laboratories and maintained under pathogen-free conditions and the veterinary care of the Animal Care Services (ACS) facility at the University of Florida.

Five groups (10 mice/group) were vaccinated in a prime-boost fashion. Briefly, mice received two intramuscular (IM) injections at 2-week intervals with 100 μg of endotoxin-free plasmid DNA *virB9-1*, *virB9-2*, or *virB10*, a mixture of the three constructs, or *pcDNA3.1*/CT-GFP empty vector. Two weeks after the last DNA immunization, the same groups received two subcutaneous (SC) injections at two-week intervals with 100 μg of their respective purified recombinant VirB9-1, VirB9-2, or VirB10 proteins, a mixture of the three recombinant proteins, or Ovalbumin (InvivoGen) in 15 μg of Quil-A adjuvant (Sergeant Adjuvants).

Two weeks after the last recombinant protein immunization, three mice from each group were randomly selected and euthanized for collection of serum samples and harvesting of their spleens to determine immune responses before challenge. Approximately 5 μl serum was also collected from the remaining seven mice per group. These remaining seven mice per group were then challenged intraperitoneally (IP) with *A. phagocytophilum* organisms isolated from 5.63 × 10^5^ HL-60 cells (90% of the cells contained morulae) that were lysed by needle aspiration through 25Ga and 30Ga needles. Blood samples to determine bacterial load were collected starting 2 days post-challenge and every other day. Mice were euthanized at day 14 post-challenge, spleens were harvested, and serum samples collected.

### Antibody responses

To determine sera specificity against *A. phagocytophilum* by indirect immunofluorescence assay (IFA), HL-60 cells (10^4^ cells per well) 80% infected with *A. phagocytophilum*/HZ were fixed with acetone for 10 min at room temperature onto 12 well Teflon-coated microscope slides (Tekdon inc). Samples were blocked by adding 20 μl of 5% bovine serum albumin (BSA) diluted in 1X phosphate-buffered saline (PBS) (Hyclone), incubated in a humidified chamber atmosphere at room temperature (RT) for 1 h, and subsequently rinsed with wash buffer (0.05% Tween 20 (Sigma-Aldrich) in 1X PBS). Sera from the three mice from each group were then combined and serially diluted 1:80 to 1:81,920. Ten μl of serum were applied to each well in duplicate for each dilution, and incubated for 1 h at RT in a humidified chamber. After incubation, the sera were removed, and the slides washed five times for 5 min. Ten μl of Alexa Fluor 568-goat anti-mouse IgG antibody (ThermoFisher Scientific) at a dilution of 1:1600 was applied to each well and incubated for 1 h at RT in a humidified chamber. The slides were washed as described above and mounted with ProLong Gold antifade reagent with DAPI (4′,6-diamidino-2-phenylindole) (ThermoFisher Scientific) for visualization of fluorescent *A. phagocytophilum* inclusions.

Antibodies from these immunized mice were assessed by SDS-PAGE and immunoblotting using equal amounts (10^8^) of host cell-free bacteria. Membranes were incubated with sera at a dilution of 1:1000. Antibody binding was detected with the secondary antibody horseradish peroxidase-goat anti-mouse IgG (Sigma-Aldrich) at a final concentration of 1:100,000 and the SuperSignal West Femto substrate (ThermoFisher Scientific) as described by the manufacturer.

Sera collected from the ten rVirB-vaccinated mice and control group, before challenge, were tested by an enzyme-linked immunosorbent assay (ELISA) to measure the level of antibodies against *A. phagocytophilum* organisms. A 384-well MaxiSorp microtiter plate (Nunc) was coated overnight at 4 °C with host cell-free *A. phagocytophilum* organisms (10^8^/well) in 50 μl of carbonate-bicarbonate buffer (Sigma-Aldrich). Next morning the wells were rinsed three times with wash buffer (1X PBS pH 7.3 MT-PBS; 150 mM NaCl, 15.9 mM Na_2_HPO_4_, 4 mM NaH_2_PO_4_ with 0.05% Tween 20) and blocked for 2 h at RT with 100 μl of 1% bovine serum albumin BSA in MT-PBS. Blocking buffer was removed, wells were washed 3X and then incubated with 1:150, 1:300, 1:600 and 1:1200 dilutions (35 μl) of the sera from all the immunized and control mice for 2 h at RT with shaking. After incubation, the wells were washed three times and reacted with recombinant protein A/G-Alkaline phosphatase (Sigma-Aldrich) at a dilution of 1:5000 for 1 h at RT with shaking. Color was developed using 4-Nitrophenyl phosphate (Sigma-Aldrich), and the optical densities of the reactions were measured using a microplate reader (Synergy HT Bio-Tek) at 405 nm. Titers were expressed as the reciprocal of the highest dilution. Results were considered positive when the signal/cutoff value was above the mean OD_405_ response from seronegative samples (*pcDNA3.1*/Ovalbumin-vaccinated mice) plus the standard deviation (SD) multiplied by 1.923 (this factor was used based on the number of negative control samples) [60].

### Cellular immune responses

Spleens were aseptically removed and transferred into 2.0 ml safe-lock tubes (Eppendorf) containing one 5 mm stainless steel bead and homogenized in a TissueLyser II apparatus (Qiagen) for 2 min at 20 Hz. The homogenate was then passed throughout a 70-μm nylon mesh (BD Falcon), resuspended in 5 ml of sterile H_2_O to lyse red blood cells, and then brought to a final volume of 20 ml by adding flow cytometry (FACS) buffer (Dulbecco’s phosphate buffered saline (Sigma-Aldrich), 2% FBS). Cells were then pelleted at 400 x g for 5 min at 4 °C, stained with trypan blue, and enumerated using a Nexcelom Bioscience cellometer. Mouse splenocytes were cryopreserved in 1.0 ml of freezing medium (90% FBS, 10% DMSO) [61].

Frozen splenic cell suspensions were thawed at 37 °C, resuspended in complete medium (CM) (RPMI 1640 medium (HyClone) supplemented with 10% FBS, 0.25% NaHCO_3_ (Sigma-Aldrich), 25 mM HEPES (Sigma-Aldrich), 1X of non-essential amino acids (Sigma-Aldrich), and 1X of penicillin/streptomycin (penicillin 100Units/mL, streptomycin 100 μg/mL (Sigma-Aldrich)) and centrifuged at 400 x g for 5 min at room temperature. Pelleted splenic cells were resuspended in CM, enumerated, and then cultured in round-bottom 96-well tissue culture plates at a concentration of 5 × 10^6^ cells/ml and stimulated with 5 μg/ml of host cell-free *A. phagocyotphilum* organisms (heat-killed at 65 °C for 10 min) or medium only (unstimulated control) in triplicate wells at 37 °C in 5% carbon dioxide (CO_2_) atmosphere. Spleen cells were harvested after 18 h of antigen stimulation followed by a four-hour incubation with 1 μg/ml ionomycin (eBioscience), 20 ng/ml phorbol 12-myristate (PMA), and 10 μg/ml of Brefeldin A (eBioscience).

For flow cytometry, cells were harvested and washed in FACS buffer and surface stained with fluorochrome-labeled antibodies specific for mouse CD4 (Alexa Fluor 700, clone: GK1.5) (eBioscience) and CD8 (Brilliant violet 510, clone: 53-6.7) (Biolegend) for 20 min on ice. In addition, intracellular cell staining was performed to detect intracellular cytokines. After cell surface marker staining, washed cells were fixed and permeabilized using an intracellular fixation and permeabilization buffer set (eBioscience) as per manufacturer’s instructions. Lymphocytes were stained intracellularly with fluorochrome-labeled antibodies specific for mouse IFN-γ (Brilliant violet 785, clone: XMG1.2, Biolegend) and IL-10 (PE, clone: JES5-16E3, eBioscience) for 20 min at room temperature. Cells were washed and resuspended in FACS buffer and 250,000 events were collected on a LSR Fortessa flow cytometer (BD Biosciences), and data analyzed using FlowJo software. The frequencies of antigen-specific T cells in the spleens from vaccinated and control mice were determined after the background staining of unstimulated cells in wells containing medium only was subtracted.

### Quantitative real-time PCR of blood

DNA isolation from mouse blood was performed using the Quick-gDNA blood microprep kit (Zymo Research) as per manufacturer’s instructions. The DNA concentration of each sample was determined using the Qubit dsDNA high sensitivity (HS) assay kit (Life Technologies) on a Qubit fluorometer (Life Technologies). Quantification of *A. phagocytophilum* genome equivalents (GE) was performed by qPCR with primers and a probe targeting the single copy gene *msp5* (Table 1). Triplicate reactions from each sample per group at each time point were used. Reactions of 20 μL containing 2 μL of genomic DNA, 1X of LightCycler 480 probe master mix (Roche), 0.4 μM forward and reverse primers and 0.2 μM of probe were used for amplification in a LightCycler 580 real-time PCR instrument (Roche) with the following conditions, 95 °C for 10 min and 45 cycles of 94 °C for 10s, and 60 °C for 30s. Ten-fold serial dilutions of the pCR-TOPO vector carrying the *A. phagocytophilum msp5* gene were used for standard curve preparation, and the *A. phagocytophilum* GE number was calculated based on the standard curve. Results were normalized based on the volume of blood collected per animal at each time point. No template control and genomic DNA extracted from the blood of unchallenged mice were used as negative controls.

### Statistical analysis

Data are expressed as Mean ± SE or SD values. One way analysis of variance (ANOVA) with Dunnett’s posttest were used to compare multiple tests groups to the control *pcDNA3.1*/Ovalbumin vaccinated group and unpaired two-tailed t test was used for comparison of two groups using Sigmaplot version 12 (Systat Software Inc., San Jose, CA), and graphics made with GraphPad Prism (GraphPad Software Inc., La Jolla, CA). Data from T cell immune responses were square-root transformed to normalize variances.

## Abbreviations

CMV: Cytomegalovirus
ELISA: Enzyme-linked immunosorbent assay
GE: Genome equivalents
HGA: Human granulocytic anaplasmosis
IFA: Immunofluorescence Assay
IFN-γ: Interferon-gamma
IL-10: Interleukin-10
SDS-PAGE: Sodium dodecyl sulfate polyacrylamide gel electrophoresis
T4SS: Type 4 Secretion System
